# Self-managed occupational therapy and physiotherapy for adults receiving inpatient rehabilitation (‘My Therapy’): protocol for a mixed-methods process evaluation

**DOI:** 10.1186/s12913-021-06463-8

**Published:** 2021-08-13

**Authors:** Sara L. Whittaker, Nicholas F. Taylor, Keith D. Hill, Christina L. Ekegren, Natasha K. Brusco

**Affiliations:** 1 Rehabilitation, Ageing and Independent Living (RAIL) Research Centre, School of Primary and Allied Health Care, Monash University, 47-49 Moorooduc Hwy, VIC 3199 Frankston, Australia; 2 La Trobe University Centre for Sport and Exercise Medicine Research, Plenty Road & Kingsbury Drive, 3086 Bundoora, Australia; 3 Eastern Health, 5 Arnold St, 3128 Box Hill, Australia; 4 Alfred Health, 55 Commercial Rd, 3004 Melbourne, Australia

**Keywords:** Process evaluation, self-management, rehabilitation, logic model, independence, intensity, occupational therapy, physiotherapy, dose

## Abstract

**Background:**

Process evaluations have been recommended alongside clinical and economic evaluations to enable an in-depth understanding of factors impacting results. My Therapy is a self-management program designed to augment usual care inpatient rehabilitation through the provision of additional occupational therapy and physiotherapy exercises and activities, for the patient to complete outside of supervised therapy. The aims of the process evaluation are to assess the implementation process by investigating fidelity, quality of implementation, acceptability, adoption, appropriateness, feasibility and adaptation of the My Therapy intervention; and identify contextual factors associated with variations in outcomes, including the perspectives and experiences of patients and therapists.

**Methods:**

The process evaluation will be conducted alongside the clinical and economic evaluation of My Therapy, within eight rehabilitation wards across two public and two private Australian health networks. All participants of the stepped wedge cluster randomised trial (2,160 rehabilitation patients) will be included in the process evaluation (e.g., ward audit); with a subset of 120 participants undergoing more intensive evaluation (e.g., surveys and activity logs). In addition, 24 staff (occupational therapists and physiotherapists) from participating wards will participate in the process evaluation. The mixed-methods study design will adopt a range of quantitative and qualitative research approaches. Data will be collected via a service profile survey and audits of clinical practice across the participating wards (considering areas such as staffing profiles and prescription of self-management programs). The intensive patient participant data collection will involve structured therapy participation and self-management program audits, Exercise Self Efficacy Scale, patient activity logs, patient surveys, and patient-worn activity monitors. Staff data collection will include surveys and focus groups.

**Discussion:**

The process evaluation will provide context to the clinical and economic outcomes associated with the My Therapy clinical trial. It considers how clinical and economic outcomes were achieved, and how to sustain the outcomes within the participating health networks. It will also provide context to inform future scaling of My Therapy to other health networks, and influence future models of rehabilitation and related policy.

**Trial registration:**

This study was prospectively registered with the Australian and New Zealand Clinical Trials Registry (ACTRN12621000313831; registered 22/03/2021, http://www.anzctr.org.au/Trial/Registration/TrialReview.aspx?id=380828&isReview=true).

## Background

Randomised controlled trials are considered the gold standard for determining the efficacy of an intervention or program. However, they do not provide context for how an intervention might be replicated in different settings, whether trial outcomes can be reproduced and what factors positively or negatively influence reported outcomes [1, 2]. The United Kingdom Medical Research Council (MRC) has recommended that researchers include process evaluations alongside clinical trials [2]. It has been argued that researchers have an ethical requirement to embed process evaluations within trials of complex interventions to provide contextual information, consider intervention fidelity, quality of implementation and possible explanations for trial results [3, 4]. A well-designed and thoroughly-reported process evaluation can provide insight into why an intervention failed unexpectedly or had unanticipated consequences and can assist in understanding how an intervention can be optimised if it is effective [2].

Process evaluations aim to capture intervention fidelity (whether the intervention was delivered as it was intended) and dose (the quantity of intervention implemented) [1]. In addition, process evaluations should enable researchers to understand how the intervention was delivered, whether any adaptations were made and its ‘reach’ (the extent to which the intended population came into contact with the intervention) [1]. It has been recommended that assumptions in the form of a logic model should be published with process evaluation protocols to show how the hypothesised mechanism/s of the intervention interact [1]. The use of a logic model in complex program analysis assists as a framework for understanding how the program theoretically works to achieve benefits [5].

This protocol paper is associated with the My Therapy Stepped Wedge Cluster Randomised Trial Protocol describing the clinical and economic outcomes [6]. The other protocol paper describes the clinical and economic evaluation of My Therapy, a self-management program designed to augment usual care occupational therapy and physiotherapy rehabilitation through the provision of additional independent practice of occupational therapy and physiotherapy exercises for completion outside of supervised therapy [6]. In a feasibility study, participants allocated to My Therapy completed an average of 100 additional minutes per week outside of structured therapy, compared to the usual care control group [6]. This feasibility study also showed a significantly higher proportion of participants in the My Therapy intervention group improved by a minimal clinically important difference (MCID) on the Functional Independence Measure (FIM), compared to the control group [6]. With no adverse events or safety concerns identified, including the delivery of the program to those with cognitive impairment, the pilot work provided sufficient evidence to support a multi-site trial of My Therapy [6]. This process evaluation protocol paper details the process evaluation which will run alongside the clinical trial of My Therapy. To our knowledge, there are no other published papers reporting a process evaluation of a self-management program in rehabilitation settings. Results of this study may be applicable to other services delivering physical rehabilitation, as well as policy makers and clinicians delivering or recommending a self-management program for adults with a range of health conditions.

The study aims to: (1) assess implementation by investigating fidelity, quality of implementation, acceptability, adoption, appropriateness, feasibility and adaptation of the My Therapy intervention; and (2) identify contextual factors associated with variations in outcomes, including understanding patient and therapists’ perspectives and experiences of My Therapy.

## Methods/design

 This study received ethics approval from Alfred Health Human Research Ethics Committee (HREC) (69,610, [Local Reference: Project 758/20]), La Trobe University HREC (758/20) and Monash University HREC (27,546), and governance approval from Cabrini Health Research Governance (11-04-03-21); Eastern Health Office of Research and Ethics (S21-004-69610), and Healthscope Research Committee.

### Design

The My Therapy trial uses a stepped wedge cluster randomised design. It consists of a trial period of 54 weeks comprising nine blocks, each of six-weeks duration. The stepped wedge cluster design has a unidirectional crossover from the usual care condition (control group receiving usual care) to the experimental condition (intervention group receiving My Therapy in addition to usual care). Block one of the My Therapy trial will see all wards remaining under the control condition. From block two, and for each subsequent block, one of the eight wards will cross over to the experimental condition. The randomisation of the ward allocation has been detailed in the My Therapy Stepped Wedge Cluster Randomised Trial Protocol describing the clinical and economic outcomes [6].

**Fig. 1:**
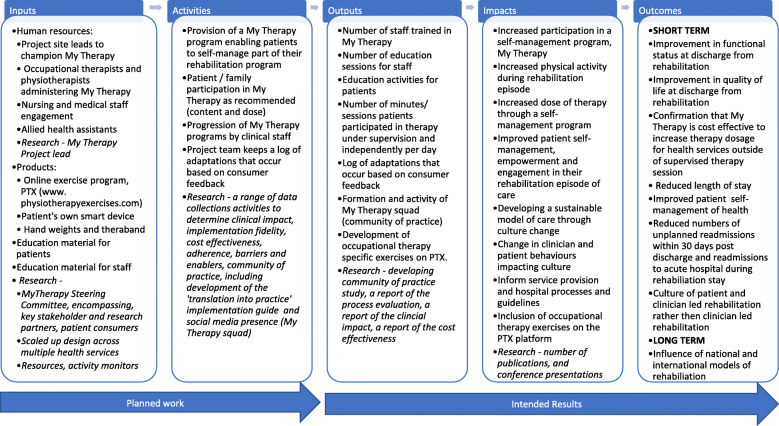
My Therapy Program process evaluation logic model

The process evaluation documented in this protocol will be conducted alongside the main clinical trial using a mixed-methods design. This protocol has been developed in accordance with the SPIRIT checklist [7] and has been based on the MRC guidelines for developing and evaluating complex interventions [1]. The intention is to report on implementation, and identify contextual factors associated with variation in outcomes [8]. The contextual outcomes considered include understanding the perspectives and experiences of patients and therapists using My Therapy. The use of the logic model assisted in conceptualising components of the My Therapy program considering inputs, activities, outputs, impacts, and outcomes. This logic model informed and guided the process evaluation protocol (Fig. 1).

### Setting

The main clinical trial and the process evaluation of My Therapy will take place in eight rehabilitation wards across two public and two private Victorian health networks (in Australia).

### Participants

Patient participants: All participants of the stepped wedge cluster randomised trial (2,160 rehabilitation patients) will be included in the process evaluation (service profile survey and ward compliance audit - Table 1); with a subset of 120 participants undergoing more intensive evaluation (e.g., surveys and activity logs), subject to eligibility. The process evaluation will include patients aged 18 years or older, with any diagnosis, who have been admitted to one of the participating wards. The evaluation of the subset of 120 patients will exclude patients with a cognitive impairment, patients without access to Medicare (Australia’s universal health care program), as well as those who do not speak English. The cognitive exclusion is due to the burden of the data collection requirements within the process evaluation and the requirement for participants to recall their experience of rehabilitation. Cognitive impairment will be determined via health professional documentation within the medical record and/or through discussion with treating allied health, medical and nursing staff. Should a person’s cognitive skills improve, for example following resolution of delirium, patients may be invited to participate.

**Table 1:** Process evaluation data collection

				Time points of data collection within each of the nine blocks					
**Data collection method**	**Aim**	**Evaluation component**	**Key Indicators**	**Week** **1**	**Week** **2**	**Week** **3**	**Week** **4**	**Week** **5**	**Week** **6**
1. Service profile survey (*n* = 2,160)	2	**Ward characteristics**: To identify contextual factors associated with variation in outcomes and to map barriers and enablers against the characteristics of each ward	Insight into staffing profile to compare the eight wards (four networks) and consideration of service changes that may impact My Therapy implementation	✓ Each block					
2. Ward compliance audit (*n* = 2,160)	1	**Staff adoption**: To measure adoption of My Therapy	Within control conditions, determine the presence of self-management programs and once wards cross over to experimental conditions determine the presence of My Therapy programs			✓ Each block			
3. Audit of medical record/timetabling (*n* = 120)	1	**Staff outcome**: To measure fidelity with maintaining usual care.	Usual care therapy dose is maintained by reviewing therapy attendance within supervised sessions				✓ In blocks 1,5 and 9		
4. Audit of PTX^a^ (*n* = ~ 80)	1	**Staff outcome**: To measure staff fidelity with, and feasibility of, prescribing and modifying My Therapy; quality of implementation; as well as patient adoption and compliance with My Therapy tasks and exercises	My Therapy program is developed by occupational therapists and physiotherapists with at least one review/amended program completed over the 7-day period. Patient adoption and compliance is indicated by patients completing their My Therapy program as recommended				✓ In blocks 5 and 9		
5. Exercise self-efficacy scale (*n* = 120)	2	**Patient reported outcomes**: To measure participants’ self-efficacy and exercise behaviours to determine confidence with My Therapy (or a self-management program that meets the defined criteria in the control conditions) and motivation to complete their exercises and activities independently.	Self-efficacy scores are determined for those patients who participate in a My Therapy Program (or self-management program)				✓ In blocks 1,5 and 9		
6. Patient survey (*n* = 120)	2	**Patient reported outcomes**: To understand the impact of My Therapy (or a self-management program that meets the defined criteria in the control conditions) on self-management and feelings of empowerment, patients’ ability to self-manage health, if My Therapy is perceived to be a joint patient-clinician led experience, acceptability, appropriateness, and the barriers and enablers to participation	Reporting qualitative data regarding patients perceptions.				✓ In blocks 1,5 and 9		
7. Activity log (*n* = 120)	1	**Patient outcome**: To measure patient fidelity with the recommended My Therapy program (or a self-management program that meets the defined criteria in the control conditions)	The My Therapy Program (or self-management program) is completed at the frequency that was recommended.				✓ In blocks 1,5 and 9		
8. Activity monitoring (*n* = 36)	1	**Patient outcome**: To measure patient fidelity with the recommended My Therapy program	Participant activity levels are reported during the My Therapy Program (or self-management program)				✓ In blocks 1,5 and 9		
9. Staff survey (*n* = 24)	1 & 2	**Staff outcome**: To measure fidelity, quality of implementation and factors influencing the implementation of My Therapy. For the staff who are working on the control wards, this is their perceptions regarding self-management programs.	Reporting qualitative data regarding therapists perceptions.			✓ In blocks 1,5 and 9			
10. Staff focus groups (*n* = 24)	1 & 2	**Staff outcome**: To measure fidelity, quality of implementation and factors influencing the implementation of My Therapy	Reporting qualitative data regarding therapists perceptions.					✓ In blocks 1,5 and 9	

Study aims: (1) assess implementation by investigating fidelity, quality of implementation, acceptability, adoption, appropriateness, feasibility and adaptation of the My Therapy intervention; and (2) identify contextual factors associated with variations in outcomes, including understanding patient and therapists’ perspectives and experiences of My Therapy

^a^For wards under experimental conditions only

Staff participants: 24 registered occupational therapists and physiotherapists from participating wards, who are involved with the My Therapy trial, will be invited to participate in the process evaluation. There are no exclusion criteria for staff participation.

### Recruitment

Patient participants: A subset of 40 patients (five from each of the eight wards) will be recruited to the process evaluation across three time points (randomisation blocks one, five and nine; total *n* = 120). Patients will be recruited using a convenience sampling approach, based on consecutive admissions over the study period. While it is expected this sampling method will recruit a diverse sample, participants will not be purposively recruited based on age or condition. A sample of size of 120 will provide sufficient power to detect a worthwhile effect (0.5) [9] on self-efficacy, with alpha = 0.05, power = 0.8, and assuming equal sample sizes in control and experimental groups. This sample size (*n* = 120) will also be sufficient to determine that 85 % of participants have the factor of interest (e.g. patient compliance with activity log) with 95 % confidence that between 78 and 92 % have the factor of interest, assuming an intra-class correlation coefficient of 0.005 and a cluster size of 15 [10].

Staff participants: A subset of eight therapists (one from each of the eight wards) will be recruited to the process evaluation across three time points (randomisation blocks one, five and nine; total *n* = 24 independent therapists). Purposive sampling will be used to target one representative from each of the participating wards during each recruitment block and to ensure inclusion of both occupational therapy and physiotherapy staff with a range of years of clinical experience. The sample of 24 therapists is expected to be sufficient to reach saturation in qualitative analyses.

### My Therapy program (the intervention)

The My Therapy program is described in detail in the My Therapy Stepped Wedge Cluster Randomised Trial Protocol describing the clinical and economic outcomes [6]. In summary, each ward will transition from the usual care condition (control group receiving usual care) to the experimental condition (intervention group receiving My Therapy in addition to usual care). My Therapy is a self-management program designed to increase dose of therapy by supporting patients to independently practice occupational therapy and physiotherapy exercises and activities outside of supervised therapy sessions. My Therapy is a partnership model based on the patient’s rehabilitation goals, abilities and willingness to participate, recommended by the treating occupational therapist or physiotherapist. Patients will be asked to complete exercises and activities selected from an online program, www.physiotherapyexercises.com (PTX). The My Therapy exercises and activities will be sent to patients via SMS or email and viewed on a ‘smart device’ (patient’s own) or printed in hard copy and provided to the patient. In Australia, 86 % of households have internet access and 91 % of those with internet access use a mobile or smart phone [11], supporting the option of ‘smart device’ use. The recommended exercises, as well as the dose (sets and repetitions) recommended, can be customised and updated regularly by the treating therapists, as appropriate.

Within the four health networks, self-management is not currently standard practice within usual care rehabilitation; it is ad hoc, clinician-dependent and varies in terms of its definition within rehabilitation [12]. To understand the presence of self-management programs within control conditions, it is essential that therapist advice or education is differentiated. For the control condition, a self-management program, should it be provided by the treating therapist, needs to meet all of the following criteria: a written program (delivered electronically or in paper format); documented by the therapist in the medical record; include a feedback mechanism to the therapist; and be actively monitored and progressed, as clinically indicated. For wards under the experimental condition, this specifically refers to provision of a My Therapy program.

### Process evaluation outcomes and data collection

The data collection approach for the process evaluation is outlined in Table 1. There will be three layers of data collection to address the objectives of the process evaluation (ward, patient and staff) using a combination of qualitative and quantitative methods. In addition, a log of My Therapy adaptations (to the program itself or to how the program is implemented) will be maintained over the nine blocks of data collection, based on iterative feedback from staff, patients and consumer representatives.

1. Ward evaluation (whole of ward)

In each of the nine blocks, a service profile survey will be completed to capture the characteristics of each rehabilitation ward (for example staffing ratios, inpatient vs. home-based ward). This will provide the opportunity to identify contextual factors associated with variation in outcomes and map barriers and enablers against the characteristics of each ward.

In each of the nine blocks, a ward compliance audit will also be completed to measure patient participation in a self-management program on a set day. The audit will capture information regarding the number of patients on the ward and the number of patients with a self-management program through staff feedback (Table 1).

2. Patient evaluation

Patient evaluation (Table 1) will be completed in blocks one, five and nine. This will include: an audit of PTX to measure patient completion (i.e., adoption) of My Therapy exercises and activities (experimental condition wards only); a patient survey, including an exercise self-efficacy scale, to understand the acceptability and impact of self-management / My Therapy, as well as the patient barriers and enablers to participation; and an activity log to measure patient fidelity with completing My Therapy tasks and other self-management exercises. A small subset of patients will also complete activity monitoring to further understand patient fidelity with the My Therapy program.

The Spinal Cord Injury Exercise Self Efficacy Scale (ESES) will be used to measure participants’ exercise behaviours and exercise self-efficacy, enabling researchers to understand the person’s confidence and ability to self-manage their My Therapy program (or self-management activities) [13]. The ESES will be completed on the final day of the activity log recording. The ESES has 10 items, each rated on a 4-point Likert scale [13]. Scoring can range from 10 to 40, with a higher score indicating higher exercise self-efficacy. Acceptable reliability and validity has been noted with the SCI population [13], and the tool has been applied to patients with neurological conditions [14], congenital heart disease [15], and chronic obstructive pulmonary disease [16]. In cases where one or more items of the ESES have missing data, but over half of the items have been completed, the average score across all completed items will be used for the missing item scores, to calculate an overall ESES score.

Twelve patients, from two of the four health services, will be asked to participate in physical activity monitoring (during blocks one, five and nine; total n = 36). The participants in block one will be under control conditions, the participants in block five will have patients under both experimental and control conditions, and participants in block nine will all be under experimental conditions. This will allow us an opportunity to compare the alternate conditions at different timepoints. Participants will be asked to wear an accelerometer-based activity monitor (activPAL, PAL Technologies Limited) on the anterior, middle thigh for 24-hour monitoring over seven consecutive days. Monitors will be placed in a ziplock bag, placed on a small piece of gauze to protect the skin and covered in waterproof dressing. For patients under the experimental conditions (My Therapy), the monitor will measure the daily number of steps and standing time, as an indicator of patient fidelity with the recommended My Therapy program. The activPAL provides a valid and reliable measure of physical activity in older adults [17]. It has detected hypothesised increases in walking during inpatient rehabilitation, providing evidence of construct validity [18].

3. Staff evaluation

A survey and a focus group will be completed with participating occupational therapists and physiotherapists. Staff will firstly complete a survey followed by participation in a focus group. The staff online survey will be based on the validated Determinants of Implementation Behaviour Questionnaire (DIBQ) [19]. The survey will be sent to staff participants by researchers via email, prior to each of the focus groups. Survey responses will assist in directing the topics discussed within the focus groups. Staff focus groups will be divided into staff in the control condition and staff in the experimental conditions to ensure there is no cross contamination of views and opinions. The focus groups will be conducted and recorded over a video conference platform. A semi-structured interview guide will include questions on barriers, enablers and acceptability of My Therapy and what could make My Therapy delivery better for future implementation. For therapists whose wards remain under usual care conditions, the questions will be reframed requesting they consider any recommended ‘self-management’ programs as defined by the set criteria.

We will also complete an audit of PTX to measure staff fidelity with prescribing and modifying My Therapy tasks and exercise in blocks one, five and nine. In addition, we will complete an audit of timetabling each block for usual care supervised therapy to capture any changes in usual care occupational therapy or physiotherapy service provision by staff (Table 1).

### Data analysis

Descriptive statistics will be used for all quantitative data, in line with recommendations by Moore, Audrey [1]. Independent group t-tests or chi-squared tests will also be considered for quantitative data as appropriate. If data is not normally distributed, non-parametric equivalent tests will be used. Study data will be collected and managed using REDCap (Research Electronic Data Capture) electronic data capture tools hosted at Monash University and managed by Helix [20, 21], and exported to SPSS for statistical analysis. All analyses will assume a significance of p < 0.05. For participants who use activity monitors, should they be discharged during the seven day period while it is intended they are wearing the activity monitor, a daily average will be calculated based on the available data with a minimum of three 24-hour days of activity data required for inclusion [22].

Qualitative data from patient surveys will be entered into REDCap and uploaded to NVivo (QSR International) for analysis. Qualitative data from staff focus groups will be transcribed and, following member-checking to confirm the transcript reflects participants’ intended meaning, will be uploaded to NVivo (QSR International), along with qualitative/free-text data from staff surveys. Themes will be identified through thematic analysis, adopting an interpretive description methodological approach [23]. The interpretative description approach allows for health researchers to explore meanings and explanations that may result in clinical implications through inductive analytic processes [23]. All qualitative data will be coded independently by two members of the research team. A third researcher will be engaged should consensus not be reached on the coding framework. Participants will have the opportunity to view the transcript of their focus group or interview to check that their views have been adequately represented. The process of member checking to achieve credibility and confirmability has been considered the ‘gold standard’ for establishing trustworthiness within qualitative research [24, 25]. The concept of trustworthiness in qualitative research has been framed to also consider transferability and dependability [26]. To address the requirements of transferability, a detailed description of the sample population will be provided in addition to providing verbatim quotations. Confirmability and dependability will also be achieved by the triangulation of both quantitative and qualitative data, and by reporting a detailed description of the research process [25]. This will enable credibility, breadth and depth of the analysis of the implementation of the My Therapy program.

## Discussion

My Therapy has the potential to improve patient and health service outcomes. The process evaluation offers a perspective to understand the clinical trial outcomes (whether they are positive or negative). Should the clinical trial report positive outcomes, this process evaluation will determine how these were achieved. By understanding how benefits were achieved, the potential for sustaining and scaling benefits are enhanced.

There are a number of strengths to the design of this study. The stepped wedge cluster randomised design of the main trial provides multiple time points for data collection, allowing for repeated data collection within the process evaluation. It also enables comparison of wards under both control and experimental conditions. Another advantage of this study design is the ability to look for improved (or deteriorating) aspects of program implementation over time. This may include potential for greater awareness and engagement as the program is implemented in the longer term, or challenges over time associated with staff turnover or staff fatigue. Employing a range of data collection strategies offers an opportunity for a richer understanding of the My Therapy program than would be provided with one method alone. A governance structure, established by the My Therapy steering committee, will assist in supporting project engagement and developing strong working relationships (which have been identified as key facilitators for success of process evaluations [1]). Furthermore, employing site coordinators for the duration of the trial will assist with the consistent implementation of the protocol across sites [1].

A limitation to this process evaluation is the exclusion of the MRC theme of ‘mechanism of impact’ [1]. However, causal pathways have been hypothesised in our logic model. It is also noted that individual wards at the four health networks will be supported to adapt the My Therapy implementation plan to meet their site-specific needs, with a risk that the wards may implement My Therapy differently to what was intended. However, to account for this potential variation, site-specific adaptation will be captured and reported in the process evaluation. There will also be close monitoring of implementation through a collaborative approach with the site co-ordinators. The convenience sampling method adopted for the sub-set of patients (n = 120) may mean there is a risk that the sample is not representative of the general rehabilitation population. However, the large sample (*n* = 120 patient participants; *n* = 24 staff participants) is expected to minimise this risk.

It is envisaged that the process evaluation will inform the development of a ‘Translation into Practice’ implementation guide which can be used to support the wider implementation of My Therapy beyond project completion, as well as sustainability, should the clinical trial results be positive. In addition, a joint patient/clinician led campaign will be formed to establish a social media presence and an online ‘community of practice’. The community of practice will further support wider implementation both within the participating health networks and outside of the participating health networks, and will include consumers, occupational therapists, physiotherapists, health service leaders, government representatives, university academics and researchers. Bringing together this diverse community of practice will ensure that there is initial peer support for new networks implementing My Therapy, and ongoing peer support as health networks ensure change is embedded into usual care practice and policy.

The process evaluation will provide context to the clinical and economic outcomes associated with the My Therapy clinical trial. Should the trial be successful, this process evaluation will provide the context to how clinical and economic outcomes were achieved, and how to sustain the outcomes within the participating health networks. It will also provide context to inform future scaling of My Therapy to other health networks, and influence future models of rehabilitation and the related policy.

## Data Availability

The datasets generated and/or analysed during the current study will not be publicly available as the Ethical Review Board approval was obtained for public sharing and presentation of data on a group-level only. However, individual-level data may be available from the corresponding author on reasonable request noting that this will require separate ethics approval for the dissemination and use of the data.

## Abbreviations

DIBQ: Determinants of Implementation Behaviour Questionnaire
MRC: Medical Research Council
NHMRC: National Health and Medical Research Council
PTX: www.physiotherapyexercises.com
SCI ESES: Spinal Cord Injury Exercise Self Efficacy Scale
